# The 2010 Cholera Outbreak in Haiti: How Science Solved a Controversy

**DOI:** 10.1371/journal.ppat.1003967

**Published:** 2014-04-03

**Authors:** Fabini D. Orata, Paul S. Keim, Yan Boucher

**Affiliations:** 1 Department of Biological Sciences, University of Alberta, Edmonton, Alberta, Canada; 2 Center for Microbial Genetics and Genomics, Northern Arizona University, Flagstaff, Arizona, United States of America; 3 Pathogen Genomics Division, Translational Genomics Research Institute, Flagstaff, Arizona, United States of America; Duke University Medical Center, United States of America

## The 2010 Earthquake and Cholera Outbreak in Haiti

On January 12, 2010, a catastrophic 7.0 magnitude earthquake struck Haiti, affecting 3,500,000 people [1], [2]. This severely damaged an already marginal public sanitation system, creating ideal conditions for outbreaks of major infectious diseases. In October 2010, nine months after the earthquake, an outbreak of cholera started, which quickly spread all across the country [3]. As of January 7, 2014, 8,534 deaths and 697,256 cholera cases have been reported by the Haitian Ministry of Public Health and Population [4]. Prior to 2010, there was no reported history of cholera in Haiti, despite devastating outbreaks in the Caribbean region in the 19^th^ century [5]. Many wondered where the cholera in Haiti came from. Two hypotheses as to its origin were presented. The climatic hypothesis argued that nonpathogenic *Vibrio cholerae*, indigenous in the coastal waters of Haiti, was given the right environmental circumstances and evolved into a pathogenic strain [6]. On the other hand, the human transmission hypothesis suggested that cholera was introduced to Haiti by individuals infected in a foreign country.

## Cholera and *V. cholerae*

**Figure 1:**
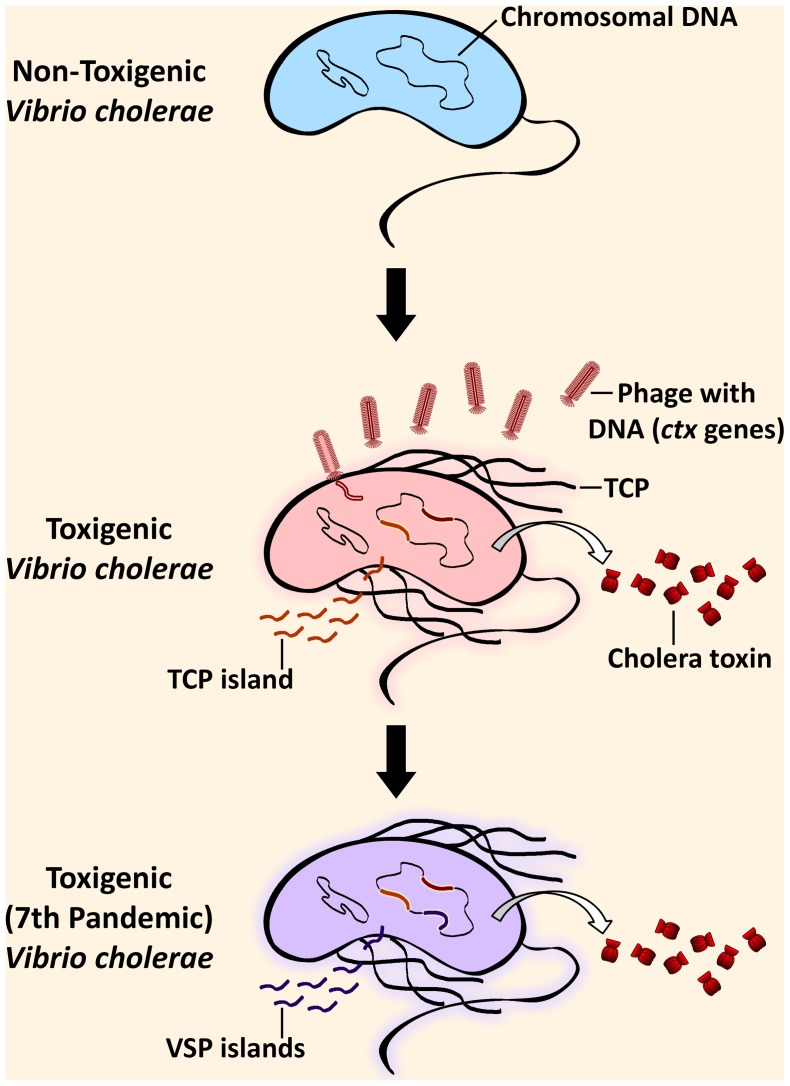
Steps in the evolution of the seventh pandemic *Vibrio cholerae*. Environmental *V. cholerae* indigenous in coastal waters can harbor genomic islands (GIs) by horizontal gene transfer, rendering it pathogenic. Pathogenesis of toxigenic (toxin-producing) *V. cholerae* critically depends on the production of the cholera toxin, which is responsible for the cholera symptoms, and the toxin-coregulated pilus (TCP). The genes for the cholera toxin (*ctx*) are from the filamentous bacteriophage, CTXφ, that has been incorporated into the genome. The genes in the TCP island encode factors necessary for the colonization of the small intestine in the human host after ingestion of contaminated water. Additionally, seventh pandemic strains are distinguishable from pre-seventh pandemic strains due to the acquisition of additional GIs, the *Vibrio* seventh pandemic (VSP) islands.

Cholera, caused by *V. cholerae*, is a disease characterized by very severe diarrhea and dehydration, which can lead to death in less than 48 hours if left untreated. Cholera is treatable through oral rehydration salt solutions, intravenous fluids, or antibiotics, depending on severity [7]. Ingestion of contaminated water is the main vehicle for human infection. The principal virulence determinant is the potent cholera toxin, encoded by the *ctxAB* genes on the bacteriophage CTXφ [8] found in toxigenic *V. cholerae* genomes. The toxin, together with other virulence factors, leads to the harmful effects of the *V. cholerae* infection (Figure 1). These auxiliary virulence factors are encoded in clusters of genes called genomic islands, which are acquired by environmental *V. cholerae* through horizontal gene transfer [9] (Figure 1). It is also important to note that infection can be asymptomatic, and these cases play a major role in the transmission of the disease [10]. *V. cholerae* is of major public health concern because of its potential to cause pandemics. Seven such pandemics have been recorded since 1817, when cholera first spread beyond the Indian subcontinent, all presumably caused by *V. cholerae* belonging to the O1 serogroup. *V. cholerae* of the classical biotype dominated the previous six pandemics and was replaced by the El Tor biotype in the currently ongoing seventh pandemic, which originated in Southeast Asia in 1961 [7]. In 1992, a new serogroup of *V. cholerae*, O139, was first identified after causing cholera epidemics in India and Bangladesh [11]. Cholera has been eliminated from industrialized countries by efficient water and sewage treatments but not in less-developed countries with poor water sanitation.

## Initial Studies Support the Human Transmission Hypothesis

**Figure 2:**
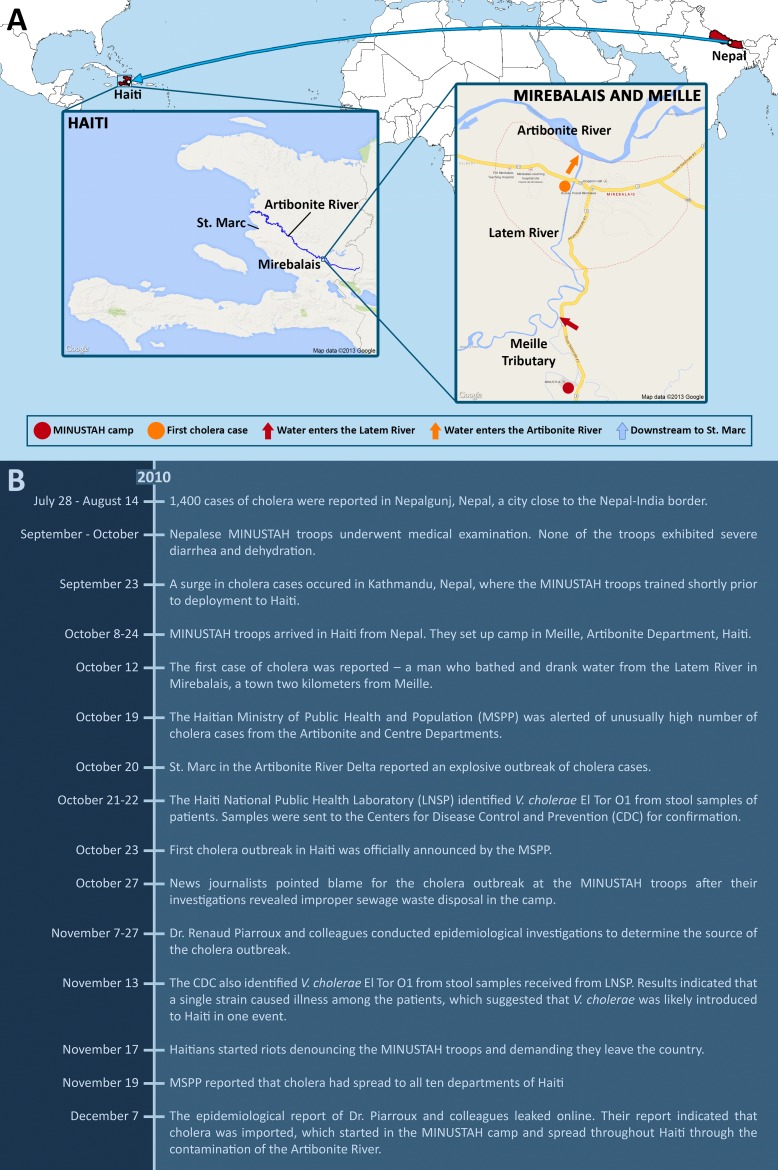
How the Haiti cholera outbreak started. (A) MINUSTAH troops from Nepal were stationed in Haiti starting on October 8, 2010, and set up camp in Meille (red circle). Improper disposal of sewage led to the contamination of the Meille tributary, which connects downstream to the Latem River (red arrow). The first case of cholera occurred on October 12 along the Latem River in Mirebalais (orange circle), 2 km north of Meille. Water from the Latem River enters the Artibonite River (orange arrow), the major river that spans across Haiti, which flows downstream to St. Marc (blue arrow). The Artibonite River played a significant role in the rapid spread of cholera. During the early onset of the epidemic, reported cases were linked to proximity with the river. (B) A chronological timeline of events involving the Haiti cholera outbreak from July to December 2010.

Rumors spread on October 27, 2010, pointing blame for the outbreak at the United Nations Stabilization Mission in Haiti (MINUSTAH) troops from Nepal who had recently set up camp in Meille, a small village 2 km south of Mirebalais (Figure 2A). This followed revelations by news reporters showing improper sewage waste disposal in the camp [12], [13].

The stool samples collected by the Haiti National Public Health Laboratory from cholera patients at the start of the outbreak were sent to the Centers for Disease Control and Prevention (CDC) for analysis. On November 13, the CDC reported that *V. cholerae* El Tor O1 was isolated from the samples and independent isolates were indistinguishable by multiple rapid phenotypic and molecular characterization methods, suggesting that a single strain caused the outbreak and was likely introduced into Haiti in one event [14].

A study by Piarroux and colleagues made use of all available epidemiological data, checking hospital records, doing field surveys, and applying statistics for spatiotemporal analysis, to trace the source and spread of the outbreak [15]. The findings of their investigation confirmed the news reporters' claims. Based on all gathered evidence, they put together a likely scenario – the MINUSTAH camp contaminated the Meille tributary with fecal matter by their unsanitary practice of sewage drainage, and the Meille tributary connects downstream to the Latem River that goes through the town of Mirebalais, the site of the first reported cholera case [16]. The Latem River eventually connects to the Artibonite River, the longest as well as the most important river that spans Haiti (Figure 2A). The movement and spread of cholera in the early onset of the epidemic was closely linked to proximity with the Artibonite River.

It had been reported that Kathmandu, the capital of Nepal, where the troops trained shortly before being stationed to Haiti, experienced a cholera outbreak on September 23 [17]. The first batch of troops arrived in Haiti on October 8 [18], and the first cholera case was reported on October 12 [16] (Figure 2B). Because none of the troops apparently exhibited symptoms of cholera during the pre-deployment medical examination, the MINUSTAH chief medical officer later revealed that no follow-up tests were done [19]. However, the absence of symptoms did not prove that the troops were *V. cholerae*-free, as they could have been infected in the days following the medical examination and prior to deployment, or they could have been asymptomatic carriers [10], [15]. Unfortunately, other than that done by the MINUSTAH, no independent testing was done of the troops to confirm the presence or absence of *V. cholerae*.

## Comparative Genomics Traced a Single Source for the Epidemic

The first molecular study on the origin of *V. cholerae* in Haiti was published on December 9, 2010 [20]. Chin and colleagues sequenced the complete genomes of two Haitian strains obtained from the outbreak, as well as epidemic strains from South America and Bangladesh, and compared them to those of epidemic-associated strains available in public databases. Comparison of single-nucleotide variations and hypervariable chromosomal elements in the genomes showed both of these Haitian strains to be genetically identical. While this is a small sample size, it was consistent with a clonal source for the outbreak. In addition, the study was able to genotype the two strains at polymorphic loci previously used for population genetic studies of *V. cholerae* [21], and this subtype had been previously observed in a broad region that included South Asia, Thailand, and Africa, but not the Americas. The study by Chin and colleagues suggested that cholera was introduced into Haiti through human transmission from a distant geographic source, most probably from South Asia (i.e., Bangladesh), although their conclusions were based upon a very limited strain analysis from both Haitian and global populations.

Two subsequent and larger genomic studies used 23 [22] and 154 [23] whole genome sequences to document the repeated historical spread of *V. cholerae* O1 from South Asia. These studies used up to nine more Haitian isolates and placed them into the context of the expanded strain genome collection. They found phylogenetic affinity between the 2010 Haitian strains and those seen in previous years from Cameroon, Bangladesh, India, and Pakistan. The Haitian isolates were nearly identical and again consistent with a single clonal outbreak. Contemporary (i.e., 2010) *V. cholerae* strains from Nepal were not included in these studies and the genomic association between Haitian and Nepalese *V. cholerae* was not differentiated from other South Asian or even African locations.

The first study to include strains from Nepal was published by Hendriksen and colleagues on August 23, 2011 [24]. It compared the genomes of 24 strains isolated from five geographic regions in Nepal (between July 30 and November 1, 2010) with ten genomes of previously sequenced *V. cholerae*, including three from Haiti. All strains from Nepal, Haiti, and Bangladesh clustered together in a single monophyletic group (i.e., they shared a common ancestor). More importantly, the three Haitian and three Nepalese strains formed a very tight subgroup within the cluster, and these were almost identical, with only one or two nucleotide difference(s) in their core genome. This study, coupled with classical epidemiology [15], [18], showed convincing evidence that cholera was introduced into Haiti from an external source, with Nepal being the most likely origin.

Despite such strong evidence supporting the human transmission hypothesis, some scientists still stood by the climatic hypothesis. A study published on June 18, 2012 by Hasan and colleagues suggested that indigenous non-O1/O139 Haitian strains were involved in the outbreak [25]. The study entailed the identification and comparison of *V. cholerae* from 81 stool samples taken from the beginning of the outbreak by traditional methods and comparative genomics. *V. cholerae* O1 was found in 48% of the samples, but more surprisingly, non-O1/O139 were identified in 21% of the samples. In addition, both O1 and non-O1/O139 strains were co-cultured in 7% of the samples, suggesting that non-O1/O139 strains may have played a role in the epidemic, whether alone or in concert with O1 strains. The authors stated that the assignment of attribution for cholera in Haiti remains controversial. However, scientists and Haitian public health officials supporting the human transmission hypothesis criticized the work, pointing out the unreliability of sampling methods [26] and that the study did not offer evidence that non-O1/O139 played a notable role in the epidemic [27].

A recent study led by scientists from the CDC and published on July 2, 2013 provided additional strong evidence to refute the climatic hypothesis [28]. Katz and colleagues sequenced the genomes of *V. cholerae* strains isolated from different time points within a two-year period since the start of the outbreak. The genomic affinity of the Haitian and Nepalese strains was reaffirmed; they were clearly distinct from isolates circulating elsewhere in the world, and there was no evidence of novel gene acquisition by horizontal gene transfer. A molecular clock was calculated, and the date of the most recent common ancestor (MRCA) was estimated to be between July 23 to October 17, 2010 (with a 95% range credibility). The human transmission hypothesis suggesting Nepalese origins would stipulate that the MRCA was in Nepal prior to deployment of the MINUSTAH troops. Additionally, this time interval encompasses the reported cholera outbreak in Nepal (September 23) [17], the arrival of the Nepalese soldiers in Haiti (October 8) [18], and the first reported cholera cases (October 12 and 17) [16], [18], supporting the time frame of the outbreak proposed by previous studies (Figure 2B) [15], [18].

## Whole Genome Sequencing as a Tool for Molecular Epidemiology

The investigations of the cholera outbreak in Haiti illustrated how traditional epidemiological investigations can be greatly enhanced by genomic sequencing and phylogenetic analysis. In this case, the analysis of hospital case records established a spatiotemporal pattern to the outbreak but failed to differentiate between the two competing hypotheses. The subsequent genomic analyses provided very strong evidence to support the human transmission hypothesis; thus, the climatic hypothesis could be rejected. These analyses, because of their ability to detect minute differences between different strains, also allowed the determination of the exact source of the outbreak. This can help enormously with prevention of future outbreaks and can also have legal implications. In a recent development, a lawsuit has been filed against the United Nations in the United States Federal Court for damages caused by the cholera outbreak [29]. With the potential for legal action, genomic analysis methods have to be very rigorous, as the judicial system will require high standards to accept them as evidence.

In retrospect, the identification of the origin of cholera in Haiti was limited by two essential factors. First was that genome sequencing was used only in the later steps of the investigation, not as a “first responder” screening method for identification of infectious agents. We are clearly entering an era where genomic or even metagenomic screening will become a part of medical diagnostics. The second limitation was the absence of a public database containing sufficient genome sequences of recurring pathogens from various geographical locations. These two elements are not out of reach, and major efforts are underway to remove both limitations [30]. For example, pipelines using current technologies to fully sequence a genome and perform key analyses for typing and identification within 24 hours are now becoming available [31]. With the price of sequencing declining, comprehensive, geographically-informed genome sequence databases of pathogens could soon be a reality.
